# Role of SNX16 in the Dynamics of Tubulo-Cisternal Membrane Domains of Late Endosomes

**DOI:** 10.1371/journal.pone.0021771

**Published:** 2011-07-06

**Authors:** Ben Brankatschk, Véronique Pons, Robert G. Parton, Jean Gruenberg

**Affiliations:** 1 Department of Biochemistry, University of Geneva, Geneva, Switzerland; 2 Institute for Molecular Bioscience and Center for Microscopy and Microanalysis, University of Queensland, Brisbane, Australia; University of Birmingham, United Kingdom

## Abstract

In this paper, we report that the PX domain-containing protein SNX16, a member of the sorting nexin family, is associated with late endosome membranes. We find that SNX16 is selectively enriched on tubulo-cisternal elements of this membrane system, whose highly dynamic properties and formation depend on intact microtubules. By contrast, SNX16 was not found on vacuolar elements that typically contain LBPA, and thus presumably correspond to multivesicular endosomes. We conclude that SNX16, together with its partner phosphoinositide, define a highly dynamic subset of late endosomal membranes, supporting the notion that late endosomes are organized in distinct morphological and functional regions. Our data also indicate that SNX16 is involved in tubule formation and cholesterol transport as well as trafficking of the tetraspanin CD81, suggesting that the protein plays a role in the regulation of late endosome membrane dynamics.

## Introduction

It is generally accepted that some long-lived lipids are not stochastically distributed in cellular membranes but are differentially distributed in subcellular compartments. The cholesterol content of the endoplasmic reticulum (ER) is low — sensing cholesterol levels in the ER regulates the expression of cholesterol-dependent gene expression — and increases from the Golgi apparatus to the plasma membrane [1]. Together with glycosphingolipids, cholesterol forms raft-like microdomains, which are believed to play a role in numerous cellular processes in the plasma membrane and other cellular membranes, including protein and lipid sorting, signaling, infection and immunity [2]. Other lipids also show restricted distributions, in particular the unconventional phospholipid lysobisphosphatidic acid (LBPA) or bis-monoacylglycerophosphate (BMP), which is abundant in late endosomes and not detected elsewhere in the cell [3]. In addition, phosphoinositides, signaling lipids that are typically very short-lived, are distributed in different cellular territories, through the concerted action of lipid kinases and phosphatases [4], [5], [6]. Typically, PtdIns(4,5)P_2_ and PtdIns(3,4,5)P_3_ are present in the plasma membrane, PtdIns(4)P in the Golgi, while PtdIns(3)P and PtdIns(3,5)P_2_ are both present in endosomes.

The human genome encodes more than 60 proteins that contain either one of two conserved motives, the FYVE or PX domain, binding phosphoinositides that are phosphorylated at the D-3 position of the inositol ring [7]. Most, if not all, PtdIns(3)P-binding proteins that have been characterized are present on early endosomal membranes, whether they contained a FYVE or a PX domain, leading to the notion that PtdIns(3)P is restricted to early endosomes. Consistently, endosomal PtdIns(3)P is mostly synthesized by the PtdIns 3-kinase VPS34, which is itself an effector of the small GTPase RAB5 that controls early endosome dynamics [8]. Conversely, FYVE or PX domain-containing proteins are expected to be restricted to early endosomes, where some may exhibit differential distributions in specialized domains or vesicle subpopulations depending on their protein partners [9], [10], [11].

In this paper, we studied the PX domain-containing protein SNX16, which was originally identified by homology with the PX domain of SNX1 [12] and is a member of the sorting nexin family [13]. We were intrigued by the observations that SNX16 is not present on early endosomes, yet membrane association depends on an intact PX domain, and is reversed by the PtdIns 3-kinase inhibitor wortmannin. We found that SNX16 is selectively enriched on tubulo-cisternal membranes of the late endosomal system, which exhibit highly dynamic properties, depending on an intact microtubule network. However, upon ectopic expression at low levels, SNX16 was hardly found on LBPA-containing vacuolar elements, presumably corresponding to multivesicular endosomes. We conclude that SNX16 together with its partner phosphoinositide define a highly dynamic subset of late endosome membranes, underscoring the notion that late endosomes are organized in distinct morphological and functional regions. Our data also indicate that SNX16 is involved in the regulation of late endosome membrane dynamics, and that this process in turn may control late endosomal cholesterol homeostasis and tetraspanin transport through the compartment.

## Results

### SNX16 is not present on early endosomes

**Figure 1:**
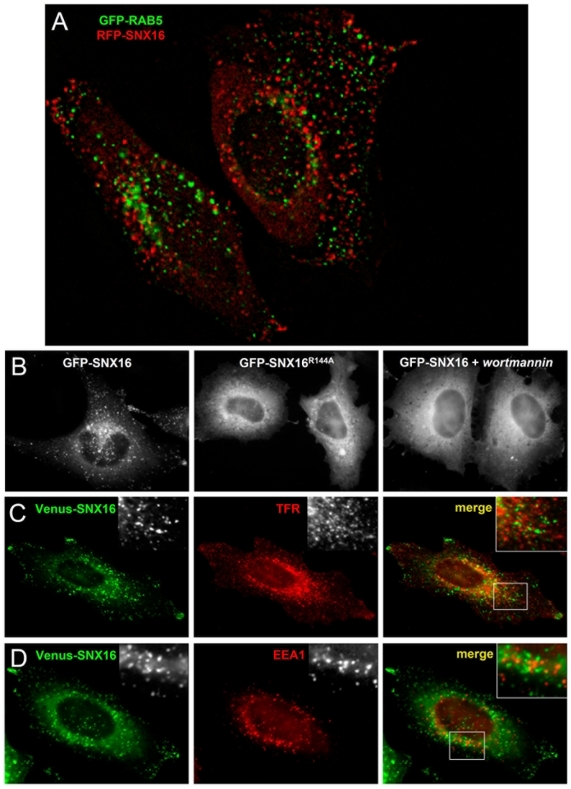
SNX16 is not present on early endosomal membranes. **A**) HeLa cells co-expressing EGFP-RAB5 and mRFP-SNX16 were analyzed by fluorescence video microscopy. **B**) HeLa cells were transfected with EGFP-SNX16 or EGFP-SNX16^R144A^ and then treated or not with 100 nM wortmannin for 30 min at 37°C, as indicated, and analyzed by fluorescence microscopy. **C**) HeLa cells were transfected with Venus-SNX16, fixed, labeled with antibodies against TFR, and analyzed by fluorescence microscopy. **D**) HeLa cells were transfected with Venus-SNX16, fixed, labeled with antibodies against EEA1, and analyzed by fluorescence microscopy.

To analyze the subcellular distribution of SNX16, cells were transfected with constructs encoding for fluorescent SNX16 fusion proteins and analyzed by light microscopy. The ectopically expressed protein showed a punctate pattern reminiscent of endosomes (Fig 1A and 1B, left) and a cytosolic pattern after treatment with the PtdIns 3-kinase inhibitor wortmannin (Fig 1B, right), suggesting that SNX16 becomes membrane-associated via interactions with PtdIns(3)P. Indeed, mutation of SNX16 Arg144 to Ala — a conserved residue of the PX domain necessary for PtdIns(3)P binding in p40^phox^ [14] — abolished membrane association (Fig 1B, middle). This is fully consistent with previous findings that SNX16 binds strictly PtdIns(3)P and no other phosphoinositide or phospholipid [12]. These observations suggested that SNX16 might be present on early endosomes that contain the bulk of PtdIns(3)P. However, to our surprise, RFP-SNX16 did not colocalize to any significant extent with GFP-RAB5 (Fig 1A). This small GTPase, which controls early endosome dynamics [15], is the best-accepted marker of early endosomal membranes and distributes to the different early endosome subpopulations that have been studied, including APPL-containing endosomes [10]. We also failed to observe significant colocalization of SNX16 fused to Venus, an improved YFP variant that allows detection of very low protein amounts [16], with any other early endosome marker tested, including the transferrin receptor (TFR, Fig 1C) and EEA1 (Fig 1D). GFP-SNX16 has been previously reported to distribute to both early endosomes and late endosomes/lysosomes [12] or to early endosomes [17], perhaps as a consequence of overexpression.

Finally, we tried to localize endogenous SNX16 with antibodies, although it had not been previously possible to detect the protein by immunofluorescence [12]. Beyond a relatively high background, these antibodies showed a punctate pattern by indirect immunofluorescence (Fig S1), reminiscent of Venus-SNX16 distribution (Fig 1), and clearly recognized SNX16, since they labeled ectopically expressed Venus-SNX16 (Fig S2). Much like SNX16, which did not overlap with GFP-RAB5 (Fig 1A), endogenous SNX16 did not colocalize with endogenous RAB5 (Fig S1A, and see below). The endogenous protein could be detected with antibodies in our cells, presumably because it is somewhat more abundant when compared to cells used in previous studies [12].

### SNX16 distributes to a subset of late endosomal membranes

**Figure 2:**
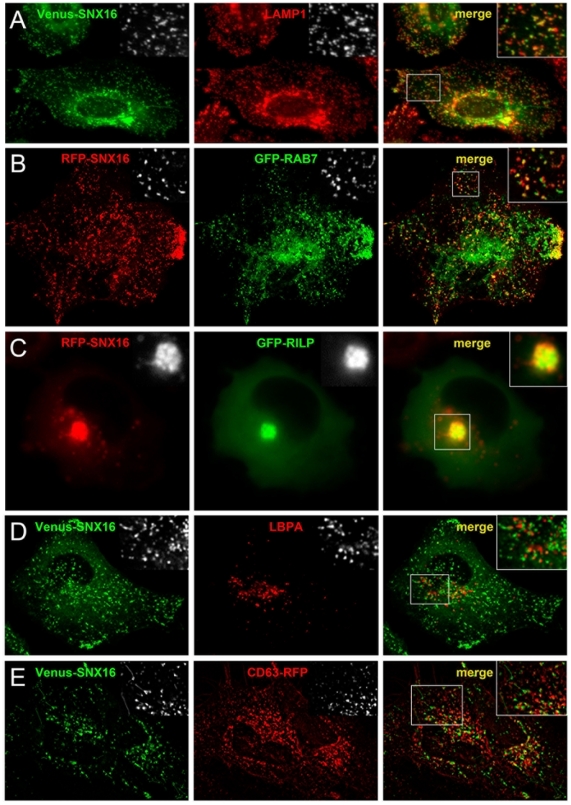
SNX16 is associated to a subset of late endosome membranes. **A**) HeLa cells were transfected with Venus-SNX16 and analyzed by immunofluorescence microscopy using antibodies against LAMP1. **B**) HeLa cells co-expressing mRFP-SNX16 and EGFP-RAB7 were fixed and analyzed by fluorescence microscopy (see also Movie S1). **C**) HeLa cells co-expressing mRFP-SNX16 and EGFP-RILP were analyzed by fluorescence video microscopy (see Movie S2). **D**) HeLa cells transfected with Venus-SNX16 were analyzed by immunofluorescence microscopy using antibodies against LBPA. **E**) HeLa cells were co-transfected with Venus-SNX16 and CD63-mRFP and analyzed by fluorescence video microscopy.

We investigated whether SNX16 was present on other subcellular organelles, but did not observe colocalization with markers of biosynthetic membranes (not show). By contrast, Venus-SNX16 showed significant colocalization with LAMP1 (Fig 2A), an abundant glycoprotein of late endosomes and lysosomes. Consistent with these data, an analysis by indirect immunofluorescence with antibodies to SNX16 showed significant colocalization of both endogenous SNX16 (Fig S1B) and Venus-SNX16 (Fig S2A–C) with LAMP1. The presence of SNX16 on late endocytic membranes was further supported by the observations that RFP-SNX16 showed significant colocalization with the GFP-tagged version of RAB7 (Fig 2B), a small GTPase present on late endosomes [15], and that Venus-SNX16 and RFP-RAB7 remained associated in time (Movie S1). To further investigate the presence of SNX16 on late endocytic membranes, we made use of the fact that overexpression of the RAB7 effector RILP causes late endosomes to cluster in the pericentriolar region where they remain immobile [18], [19]. Typically, RFP-SNX16 co-clustered with GFP-RILP (Fig 2C) within structures that remained paralyzed in the pericentriolar region (Movie S2), and did not contain the early endosomal marker EEA1 (Fig S3B). Moreover, beyond the background of the antibody, GFP-RILP overexpression clearly caused endogenous SNX16 to co-cluster with GFP-RILP and with LAMP1 (Fig S3A). Altogether these observations demonstrate that SNX16 is present on late endosomal membranes containing LAMP1 and RAB7.

**Figure 3 pone-0021771-g003:**
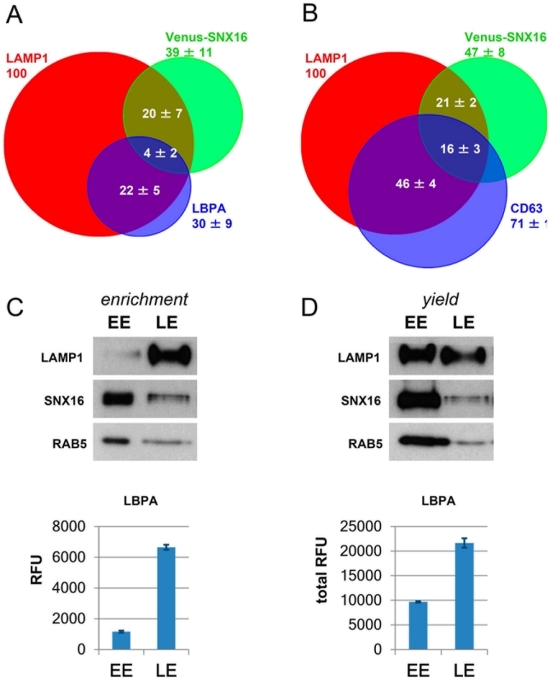
Analysis of SNX16 distribution by fluorescence microscopy and fractionation. **A–B**) HeLa cells transfected with Venus-SNX16 were labeled with antibodies against LAMP1 and LBPA (A) or LAMP1 and CD63 (B), and analyzed by confocal microscopy. The distribution of Venus-SNX16 under low expression conditions, LAMP1, and LBPA (A) or Venus-SNX16, LAMP1, and CD63 (B) was quantified after 3D image reconstruction using Imaris software (error bars indicate STDEVA). The data are expressed as the percentage of LAMP1, which co-distributes with the indicated marker. **C–D**) Untransfected BHK cells were homogenized and a post-nuclear supernatant (PNS) was prepared. The PNS was fractionated by floatation using a well-established step sucrose gradient [23]. Early (EE) and late (LE) endosome fractions were collected and analyzed by SDS gel electrophoresis and western blotting with antibodies against LAMP1, SNX16 or RAB5, or by ELISA with antibodies against LBPA. In (C), the gels were loaded with equal amounts of protein (2.5 µg), as were the wells in the ELISA analysis (5 µg), to visualize enrichment of the corresponding markers in the fractions. RFU: relative fluorescence units. In (D), the gels were loaded with equal volume (1/3 of the total fraction) to visualize the yields of the corresponding markers in the fractions. In the LBPA analysis, yields were calculated from the quantification of the ELISA data (total RFU).

To investigate SNX16 distribution in more detail we quantified the colocalization of Venus-SNX16 with LAMP1 after 3D image reconstruction of confocal sections with Imaris software. This analysis revealed that approximately half of the Venus-SNX16 structures also contained LAMP1, while LAMP1 showed a broader distribution with ≈20% present in SNX16-containing membranes (Fig 3A–B). Surprisingly, we observed little colocalization of Venus-SNX16 with the late endosome phospholipid LBPA (Fig 2D, quantification in Fig 3A), while LBPA itself showed extensive colocalization with LAMP1 (Fig 3A), as expected [20]. LBPA is abundant in the multivesicular regions of late endosomes and is not detected elsewhere in the cell [20], raising the possibility that multivesicular late endosomes do not contain significant amounts of SNX16. Consistent with this notion, the tetraspanin CD63, which is also abundant in multivesicular late endosomes containing LBPA [21], [22], showed only modest colocalization with SNX16 (Fig 2E, quantification in Fig 3B), much like with LBPA. CD63, however, showed extensive colocalization with LAMP1 (Fig 3B). We conclude that, while LBPA, CD63 and SNX16 are all present in LAMP1- and RAB7-containing late endosomes, SNX16 distributes to membrane regions or elements that are largely distinct from those containing LBPA and CD63.

The notion that SNX16 and LBPA distribute to different membrane domains or subcompartments was strengthened considerably by observations that membranes containing either marker exhibited different physical properties in sucrose gradients. After subcellular fractionation, the small GTPase RAB5 and LBPA were enriched, as expected [23], in early and late endosome fractions, respectively (Fig 3C). Surprisingly, SNX16 co-purified with early endosomes containing RAB5 and not with LBPA-containing late endosomes — and the protein had been previously found in early endosome fractions [12]. Presumably, LBPA-containing endosomes exhibit higher buoyancy on gradients (Fig 3) because of the higher lipid to protein ratio of this multivesicular compartment [22]. While LAMP1 did not co-purify with RAB5, a very significant fraction of the total LAMP1 amounts (≈50%) was found in early endosomal fractions together with SNX16 (Fig 3D), presumably corresponding to the membranes that contained both LAMP1 and SNX16 but not LBPA (total amounts of LBPA are much lower in early endosomal fractions than total LAMP1; Fig 3D). From this analysis, we can conclude that SNX16-containing membranes co-fractionate mostly with early endosomes and not with the multivesicular late endosomes. Conversely, we can also conclude that late endosomal membranes containing LAMP1 can be separated into at least two subpopulations with different physical properties on gradients, SNX16-containing membranes and multivesicular late endosome elements containing LBPA and tetraspanins.

### Late endosome tubulo-cisternal regions

**Figure 4:**
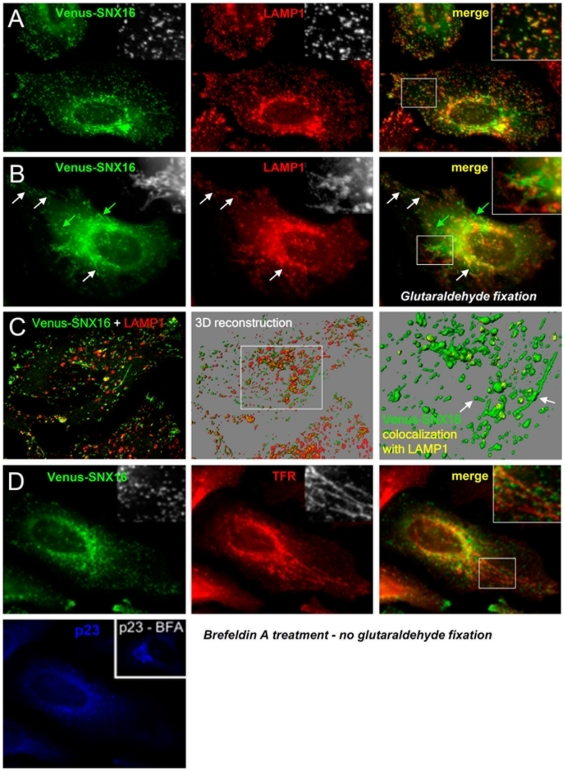
SNX16 distribution after glutaraldehyde fixation, and after brefeldin A treatment. **A–B**) HeLa cells transfected with Venus-SNX16 were fixed with paraformaldehyde (A) or glutaraldehyde (0.3%) and paraformaldehyde (3%) for 50 min (B) and analyzed by immunofluorescence microscopy using antibodies against LAMP1. Green arrows point at Venus-SNX16-positive tubules without detectable LAMP1, white arrows point to LAMP1- and SNX16-containing tubules. **C**) The left panel shows a confocal section of a cell expressing Venus-SNX16 and labeled for LAMP1 (fixation as in B). The middle panel shows a 3D reconstruction of the corresponding confocal stack with Imaris software, and the right panel shows a magnification of the boxed region, displaying only Venus-SNX16 and its colocalization with LAMP1. White arrows point out the presence of LAMP1 at discrete sites of Venus-SNX16-decorated tubules. **D**) HeLa cells transfected with Venus-SNX16 were treated with brefeldin A (5 µg/ml for 30 min) prior to fixation with paraformaldehyde and analyzed by immunofluorescence microscopy using antibodies against TFR and the *cis*-Golgi protein p23. The insert in the p23 panel shows the characteristic ribbon-like distribution of p23 in control cells without brefeldin A.

Since membranes containing SNX16 exhibit physical properties that are different from multivesicular late endosomal membranes, we wondered whether their appearance was also different. The analysis of living cells by fluorescence video microscopy (Fig 2E and see also below) suggested that a significant portion of SNX16 is present on late endosomal tubules. To gain more insight into the structures that contain SNX16, cells expressing the Venus-tagged protein were fixed with glutaraldehyde to better preserve the ultrastructure, as is the case during sample preparation for electron microscopy. Indeed, the ultrastructure of organelles, in particular membrane tubules that are notoriously fragile, is not well preserved after fixation in paraformaldehyde only. After fixation in glutaraldehyde, SNX16 and LAMP1 overlapped significantly, although some LAMP1-positive structures were devoid of SNX16 (Fig 4B) much like in paraformaldehyde-fixed cells (Fig 4A). Interestingly, SNX16 was also found within long tubulo-cisternal elements that often extended over 1–2 µm (Fig 4B and C). Moreover, it appeared that, while SNX16-positive tubules frequently contained LAMP1 (Fig 4B, white arrows), some were devoid of LAMP1 (Fig 4B, green arrows, and inset), suggesting that SNX16-containing tubules can be heterogeneous in composition. Confocal microscopy analysis and 3D reconstruction with Imaris software revealed that tubules decorated by Venus-SNX16 often contain LAMP1 (Fig 4C, right) and sometimes CD63 (not shown) at discrete sites (arrows in Fig 4C), and not all along the tubules. Since recycling endosomes also exhibit a tubular morphology [24], [25], we investigated whether the SNX16-positive tubules that were devoid of LAMP1 originated from recycling endosomes. Even in the absence of glutaraldehyde fixation, brefeldin A causes a dramatic tubularization of early/recycling endosomes containing the transferrin receptor [26] (Fig 4D). Yet, the drug did not have any effect on SNX16 distribution, demonstrating that the SNX16 tubules that did not contain detectable levels of LAMP1 were not part of the early/recycling endosome system. The effects of brefeldin A were confirmed by labeling for p23, which re-distributed from its characteristic *cis*-Golgi pattern (Fig 4D, bottom inset) to discrete perinuclear punctae (Fig 4D), reminiscent of the ER-Golgi intermediate compartment [27].

**Figure 5:**
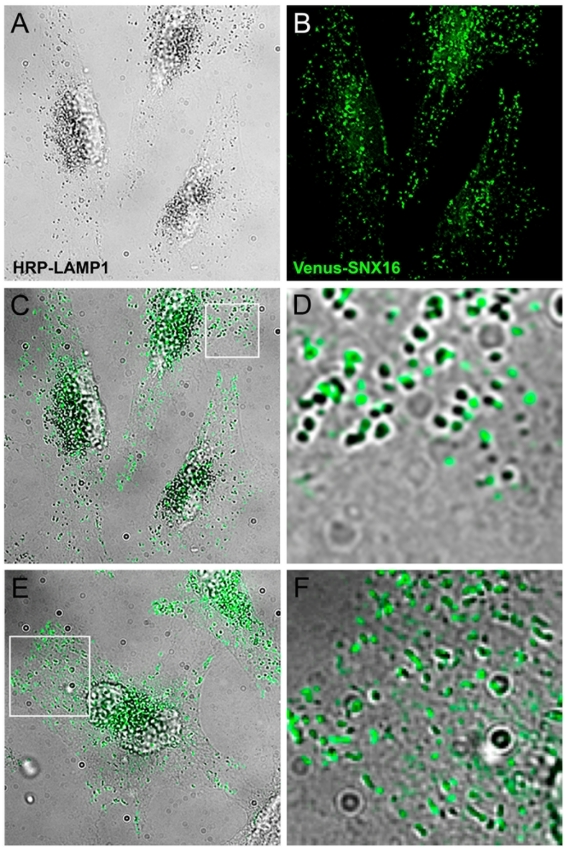
HRP-LAMP1 and SNX16 distribution on tubular and vesicular late endosomes. **A–F**) HeLa cells co-transfected with Venus-SNX16 and HRP-LAMP1 were processed as described in [28], [29]. Briefly, cells were chased with 1 mM DDT for 30 min, to ensure proper HRP-LAMP1 localization [28]. Prior to fixation, HRP-LAMP1 was revealed cytochemically with the DAB reaction and cells were permeabilized; each treatment was for 30 min at 4°C under physiological osmolarity conditions [29]. Samples were analyzed by phase contrast microscopy to reveal HRP-LAMP1 (A) and by fluorescence microscopy to reveal Venus-SNX16 (B). Panel C shows the merged image of A) and B), and panel D a high magnification view of the region boxed in C). In E), an example of a cell is shown where Venus-SNX16 and HRP-LAMP1 colocalize on numerous tubules (magnification in F).

These observations indicate that SNX16 tubules themselves may be somewhat heterogeneous in composition and may contain varying amounts of LAMP1. To analyze the distribution of SNX16 in more detail, cells were co-transfected with HRP-LAMP1 [28] and Venus-SNX16. After fixation, HRP-LAMP1 is easily revealed cytochemically using DAB as a substrate [28], [29] (Fig 5A). As expected from our immunofluorescence observations (Fig 2 **–** [Fig pone-0021771-g003] 4), we found that a significant portion of SNX16 colocalized with LAMP1 (Fig 5). In addition, this analysis also revealed that SNX16-positive structures that did not contain detectable levels of LAMP1 were frequently observed in close apposition to — and often in continuity with — LAMP1-containing structures (high magnification view in Fig 5D). Moreover, SNX16 and HRP-LAMP1 were often found together on tubular profiles (Fig 5E and F).

### Dynamics of late endosome tubules containing SNX16

**Figure 6:**
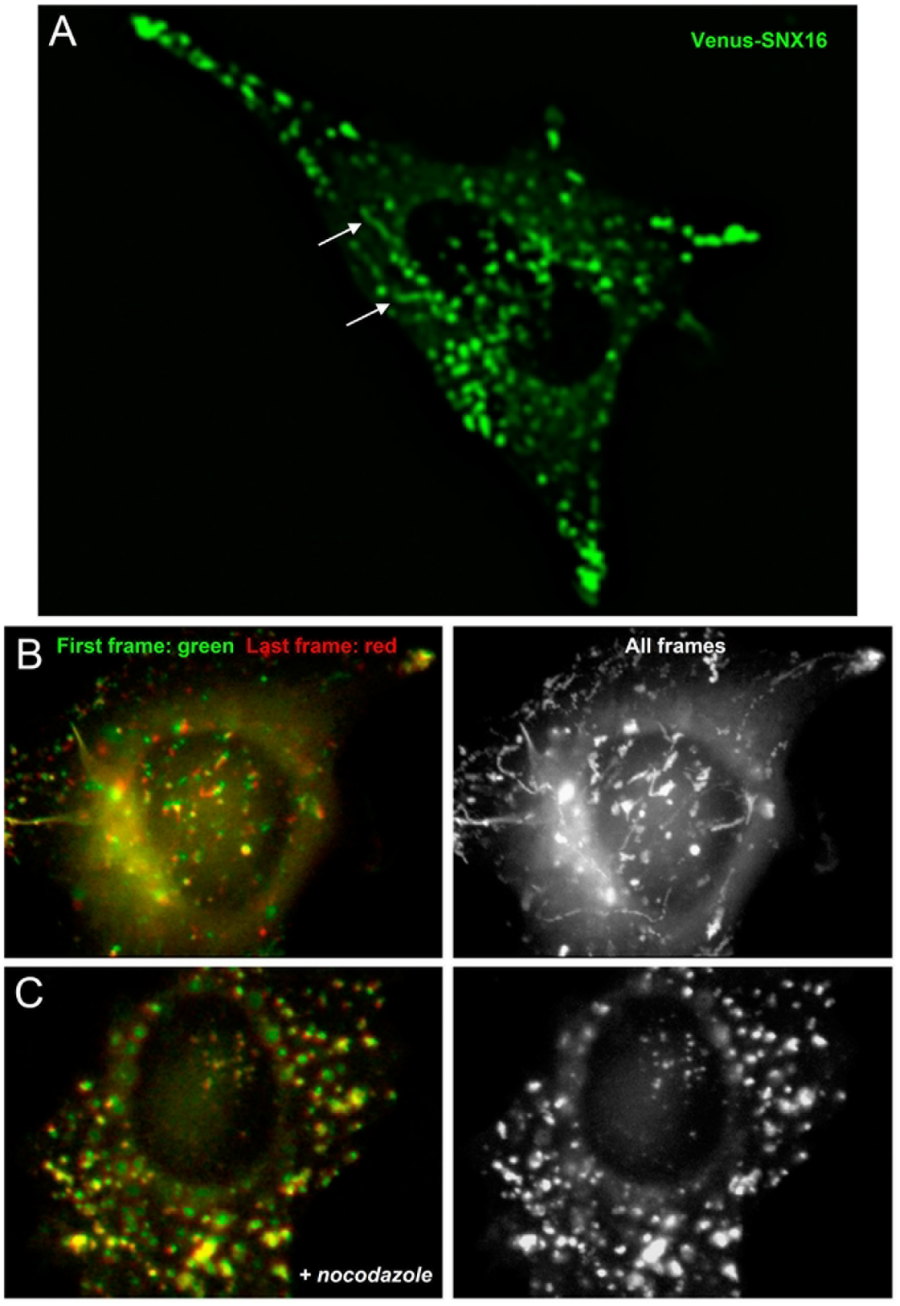
Motility of Venus-SNX16-containing endosomes depends on microtubules. **A**) HeLa cells transfected with Venus-SNX16 were analyzed by fluorescence video microscopy. Panel (A) shows a frame of Movie S3, which illustrates the dynamic tubulo-cisternal elements containing SNX16. Arrows point at highly dynamic SNX16-positive tubules. **B–C**) HeLa cells transfected with EGFP-SNX16 were pretreated (C) or not (B) with 10 µM nocodazole for 2 h, and analyzed by time-lapse video microscopy for 30 sec in the presence (C) or absence (B) of nocodazole. In the left panels, the first (green) and last (red) frames were color-coded and superimposed. The presence of each color indicates that vesicles moved while yellow shows that they remained motionless. All frames superimposed without color-code shown in the right panels feature the trajectories of the corresponding vesicles.

**Figure 7:**
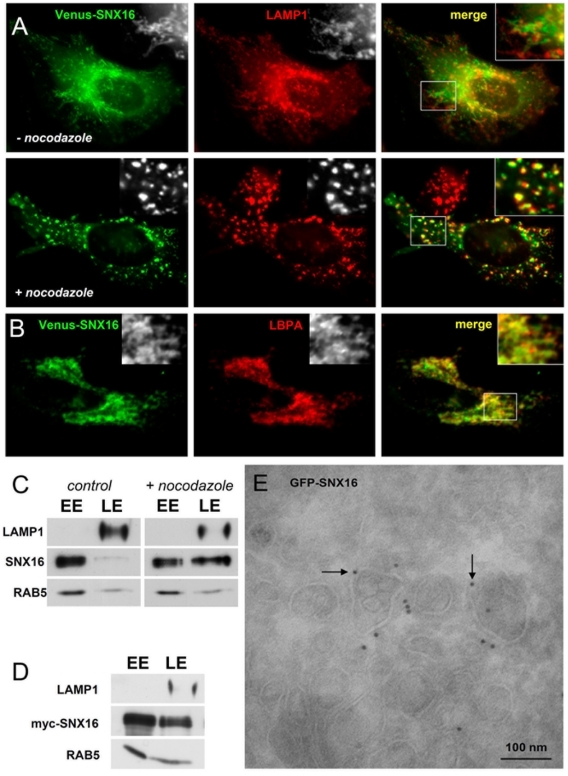
SNX16 distribution in the absence of polymerized microtubules, and re-localization to LBPA-containing multivesicular late endosomes upon overexpression. **A**) HeLa cells transfected with Venus-SNX16 were treated or not with 10 µM nocodazole to depolymerize the microtubules as in Fig 6, fixed in 0.3% glutaraldehyde and 3% paraformaldehyde, and analyzed by immunofluorescence microscopy using antibodies against LAMP1. **B**) HeLa cells overexpressing Venus-SNX16 were analyzed by immunofluorescence microscopy using antibodies against LBPA. **C**) BHK cells treated or not with nocodazole as in (A) were fractionated as in Fig 3. Early (EE) and late (LE) endosome fractions were analyzed by SDS gel electrophoresis and western blotting using the indicated antibodies. Gels were loaded with equal amounts of protein. **D**) BHK cells overexpressing myc-SNX16 were fractionated and analyzed as in (C). **E**) EGFP-SNX16-overexpressing cells were processed for cryosectioning and labeled with anti-GFP antibodies, as described [56], [57]. Scale bar is 100 nm.

The nature of SNX16-containing tubular elements became apparent when Venus-SNX16 was analyzed by time-lapse video microscopy (Fig 6A and Movie S3). The protein was primarily found in distinct elements with a characteristic tubulo-cisternal morphology (arrows in Fig 6A) similar to those observed in glutaraldehyde-fixed cells (Fig 4B–C), which distributed across the entire cell cytoplasm and did not show preferential motion towards the perinuclear region (Fig 6A and Movie S3), in contrast to the characteristic centripetal motion of endosomal vesicles containing internalized tracers. These elements aligned on microtubule tracks (not shown) and exhibited high bidirectional motility (Movie S3) that required the presence of polymerized microtubules (Fig 6B–C). Indeed, in the absence of a polymerized microtubule network, SNX16-positive structures exhibited only Brownian-like motion. Moreover, tubules disappeared after nocodazole treatment, even when analyzed in glutaraldehyde-fixed cells (Fig 7A), and SNX16 collapsed onto vesicles which remained immobile (Fig 6C). In addition, microtubule depolymerization increased SNX16 colocalization with LAMP1 by light microscopy (Fig 7A). Microtubule depolymerization also increased SNX16 co-purification with LBPA, which is present in multivesicular late endosomes with low buoyancy after subcellular fractionation (see Fig 3), without affecting early and late endosomal markers (Fig 7C). Presumably, tubule formation no longer occurred after drug treatment, leading to SNX16 accumulation on vesicular late endosomes.

Altogether, these observations further support the notion that SNX16 distributes to specialized regions of late endosomal membranes and indicate that microtubules not only support the motility of SNX16-containing tubules but also their biogenesis [30]. We conclude that late endosomes contain elements with different composition, dynamic characteristics and physical properties on gradients, and that SNX16 colocalizes with LAMP1 in highly dynamic tubulo-cisternal regions of the late endosome, which typically lack the markers of multivesicular late endosomes LBPA and CD63.

### SNX16 overexpression interferes with the dynamics of late endosomal tubules and trafficking through the compartment

**Figure 8:**
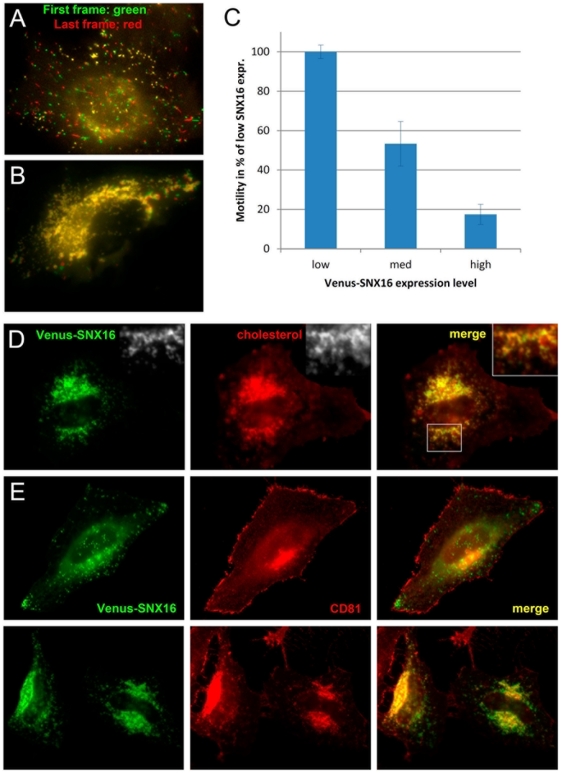
Overexpressed SNX16 inhibits late endosomal dynamics, and interferes with cholesterol and CD81 trafficking. **A–B**) HeLa cells expressing either low (A; Movie S4) or high (B; Movie S5) levels of Venus-SNX16 were analyzed by time-lapse video microscopy for 30 sec. The first (green) and last (red) frames were color-coded and superimposed, as in Fig 6B–C. **C**) The motility of Venus-SNX16-positive structures, depending on SNX16 expression levels (including medium SNX16 expression), was quantified and expressed as a percentage of the motility observed at low Venus-SNX16 expression (error bars indicate SEM). **D**) After Venus-SNX16 overexpression, HeLa cells were analyzed by fluorescence microscopy using filipin to reveal the distribution of cholesterol. **E**) CD81 distribution was analyzed by fluorescence microscopy in cells with low Venus-SNX16 (upper panels) or high Venus-SNX16 (lower panels) expression levels.

Biocomputing analysis does not predict the presence of a BAR domain that senses or induces membrane curvature in SNX16, in contrast to other members of the SNX family [31]. But SNX16 contains a predicted coiled-coil domain reminiscent of a BAR domain. A hallmark of BAR-containing proteins is their capacity to induce membrane tubulation upon overexpression [32], [33]. However, SNX16 overexpression did not increase membrane tubulation (Fig 7B), in marked contrast to SNX1 and other BAR proteins, but caused the opposite effects. SNX16-positive structures appeared clustered upon overexpression in the perinuclear region and SNX16 was no longer observed in tubules across the cell cytoplasm (Fig 7B). Moreover, while SNX16 expressed at low amounts did not colocalize with LBPA to any significant extent (Fig 2 and 3), overexpression redistributed a significant portion of SNX16 to LBPA-positive perinuclear endosomes (Fig 7B), and increased SNX16 co-purification with LBPA-containing late endosomes after fractionation (Fig 7D). Strikingly, overexpressed GFP-SNX16 was found associated with multivesicular profiles in electron micrographs (Fig 7E). In addition, an analysis by time-lapse video microscopy revealed that the motility of endosomes in cells overexpressing Venus-SNX16 (Movie S5) was significantly reduced when compared to cells containing low levels of Venus-SNX16 (Movie S4). To illustrate this effect, we color-coded the first and last frames of the movies in green and red, respectively. While green and red structures were readily visible in cells expressing low levels of SNX16 (Fig 8A), indicating that endosomes had moved during the sequence, mostly yellow structures were observed after overexpression (Fig 8B). Quantification of these sequences showed that the motility of endosomes containing SNX16 was reduced approximately 5-fold (Fig 8C) in cells expressing high levels of Venus-SNX16 (as in Fig 8B), when compared to cells expressing low levels (as in Fig 8A). We thus conclude that, much like after microtubule depolymerization, SNX16 overexpression inhibits the biogenesis of late endosomal tubules, leading to an accumulation of SNX16 on the vesicular portions of late endosomes, which contain LBPA and are abundant in the perinuclear region.

Others and us have shown that late endosomes play a crucial role in the transport of LDL-derived cholesterol [34], [35], [36]. Cholesterol can be conveniently detected by fluorescence microscopy using the cholesterol-binding drug filipin, which emits in the UV range [37], and is normally present at low (below detection) levels in late endosomes [3], [30], [38]. By contrast, in ≈100% of the cells that overexpressed Venus-SNX16, cholesterol showed a striking accumulation in late endosomes containing SNX16 (Fig 8D) and other late endosomal markers (not shown). This accumulation is reminiscent of the cholesterol storage disorder Niemann-Pick type C [3]. We were not able to investigate in a conclusive manner the effects of SNX16 depletion with the siRNAs that we have tested. However, it seems fair to conclude from our overexpression data that SNX16, which distributes to a highly dynamic subset of tubulo-cisternal late endosome membranes, plays a role in late endosomal dynamics and thereby regulates the trafficking of LDL-derived cholesterol in the endosomal system. Moreover, the tetraspanin CD81, which traffics through multivesicular endosomes [21], is normally present on the cell surface [39], [40], where it can readily be detected (Fig 8E, upper panels). Much like cholesterol accumulation, in all cells that overexpressed Venus-SNX16, CD81 was depleted from the cell surface and redistributed to late endosomes containing SNX16 (Fig 8E, lower panels) and LBPA (Fig 7B). Thus, our data, in agreement with our previous observations on endosome-to-cytosol transport of viral nucleocapsids [41], indicate that cholesterol transport and CD81 trafficking through late endosomes are inhibited by excess SNX16.

## Discussion

In this paper, we studied the PX domain-containing protein SNX16, which is a member of the sorting nexin family. We find that SNX16, in contrast to other PtdIns(3)P-binding proteins, is not present on early endosomes, yet membrane association depends on an intact PX domain, and is reversed by the PtdIns 3-kinase inhibitor wortmannin. SNX16 distributes to tubulo-cisternal elements that are part of the late endosomal membrane system, and not to multivesicular endosomes that typically contain LBPA. In a previous study, SNX16 was found in both early and late endosome fractions [12]. This observation agrees nicely with our findings that SNX16 is restricted to a subpopulation of tubulo-cisternal late endosomal membranes that co-purify with early endosomes. We find that membranes containing SNX16 are highly motile, and that these dynamic properties require polymerized microtubules. However, when present in excess, we also find that SNX16 distribution becomes shifted towards multivesicular elements containing LBPA, with a concomitant disappearance of the tubulo-cisternal elements.

Overexpressed SNX16 was previously found on early endosomes and was reported to stimulate EGF receptor degradation [17]. However, others [12] and us (not shown) did not observe such effects, perhaps suggesting that they resulted from high level of SNX16 overexpression. We find that the accumulation of SNX16 in LBPA-containing late endosomes — and the inhibition of tubule formation — is accompanied by a Niemann-Pick Type C-like accumulation of cholesterol in SNX16-containing late endosomes and a re-distribution of the tetraspanin CD81 to these SNX16-containing elements. Presumably, excess SNX16, by interfering with endosome membrane dynamics, causes cholesterol accumulation, which in turn interferes with sorting and trafficking of CD81 molecules in transit through the compartment, leading to their accumulation. Similarly, we had found that excess cholesterol causes the accumulation of CD63, another member of the tetraspanin family, in the late endosomes of endothelial cells [42]. Moreover, we also found that endocytosed antibodies to LBPA phenocopy the cholesterol accumulation observed in Niemann-Pick Type C cells [3], without interfering with EGF receptor degradation [43] — an effect reminiscent of overexpressed SNX16. Altogether, our data also fit nicely with our previous observations that, during vesicular stomatitis virus infection, excess SNX16 inhibits the delivery of viral RNA to the cytoplasm, presumably by preventing the back-fusion of intra-endosomal vesicles containing the viral RNA with the limiting membrane [41] — a process that is also inhibited by cholesterol accumulation in late endosomes [41], [44]. We thus conclude that SNX16, together with its partner phosphoinositide, define a highly dynamic subset of late endosome membranes, and that excess SNX16 interferes with this distinct morphological and functional region and thereby affects cholesterol and CD81 trafficking through the compartment in a selective manner.

SNX16 localization is surprising, since it contains a PX domain that binds 3-phosphorylated inositides necessary for SNX16 membrane association, and since it selectively binds PtdIns(3)P [12]. Yet, SNX16 distributes to late endosomal membranes. In addition to PtdIns(3,4,5)P_3_ at the plasma membrane, mammalian cells contain two other 3-phosphorylated inositides in the endocytic pathway. PtdIns(3)P has only been found on early endosome membranes, where the bulk of PtdIns(3)P is synthesized [8], and all proteins that bind PtdIns(3)P that have been characterized to date are present on early endosomal membranes. Mammalian cells also contain PtdIns(3,5)P_2_, a phosphoinositide that accumulates under hypertonic stress [45] and may be involved in autophagy [46]. The steady state amounts of this lipid in the absence of stress are very low, and its precise localization is debated. PtdIns(3,5)P_2_ is synthesized from PtdIns(3)P via the PtdIns(3)P 5-kinase PIKFYVE, which is itself a PtdIns(3)P-binding protein containing a FYVE domain [45]. The distribution of PIKFYVE itself is also a matter of debate. The current view is that PIKFYVE becomes associated to early endosomes [47], [48], [49], but may also be transported elsewhere [45]. However, knockdown of PIKFYVE had no effect on SNX16 distribution (not shown). It thus seems reasonable to conclude that SNX16 binds a specific late endosomal pool of PtdIns(3)P via its PX domain.

One possible explanation of our findings is that SNX16 becomes associated to early endosomes via PtdIns(3)P and then remains endosome-associated during transport to late endosomes, where SNX16 may accumulate via protein-protein interactions. However, we did not detect SNX16 on early endosomes, including under conditions that interfere with early-to-late endosome transport, e.g. after microtubule depolymerization. An alternative explanation is that SNX16 interacts on late endosomes with unknown protein partners. Indeed, deletion of the coiled-coil domain leads to decreased late endosomal localization and increased association with early endosomes [12] (not shown). Finally, if SNX16 interacts with PtdIns(3)P on late endosomes, these PtdIns(3)P molecules may be synthesized locally by PtdIns 3-kinase effectors of the late endosome small GTPase RAB7 [50]. One may also envision that late endosome PtdIns(3)P is derived from a pool originally synthesized on early endosomes and incorporated into intralumenal vesicles [51], which may eventually be released on the late endosome limiting membrane via back-fusion of intralumenal vesicles [36]. In any case, whether PtdIns(3)P is synthesized on early or late endosomes or whether it is released by back-fusion, it is attractive to propose that PtdIns(3)P-containing sites give rise to nascent tubules upon SNX16 recruitment and further stabilization by protein-protein interactions. Overexpression of SNX16 may interfere with the process by titrating out components that are necessary for tubule biogenesis, including perhaps PtdIns(3)P itself. This situation may inhibit the transport of cholesterol by reducing the dynamic properties of endosomal membranes, leading to CD81 re-distribution from the cell surface to LBPA-containing multivesicular late endosomes and NPC-like cholesterol accumulation. In turn, cholesterol accumulation may inhibit the delivery of vesicular stomatitis virus RNA to the cytoplasm during viral infection, without interfering with transport to the lysosomes [12] (not shown), as observed previously [43].

This scenario is attractive, since it provides a simple framework for the regulation of late endosome membrane dynamics. Indeed, late endosomal membranes at steady state undergo concomitant deformation in two opposite directions, towards the endosome lumen during intralumenal vesicle biogenesis and towards the cytoplasm during the formation of SNX16-containing tubules. Both processes must be controlled and integrated to ensure that membrane homeostasis is maintained. Given the key role of phosphoinositides in endosome dynamics, it is attractive to speculate that such homeostatic process is under the control of PtdIns(3)P signaling via specific effectors, including SNX16 in late endosomes.

## Materials and Methods

### Cells, antibodies, reagents and constructs

HeLa and BHK cell maintenance was described [52], as was the mouse monoclonal anti-LBPA antibody [20]. We are very grateful to Reinhard Jahn (Göttingen, Germany) for the mouse monoclonal antibody against RAB5, and to Wanjin Hong (Singapore, Singapore) for rabbit polyclonal antibodies against SNX16. Mouse monoclonal anti-CD63 (1B5) was a kind gift of Mark Marsh (London, UK). Rabbit polyclonal anti-p23 was described previously [27]. We also used mouse monoclonal antibodies against transferrin receptor (Zymed Laboratories, South San Francisco, CA), rabbit polyclonal anti-EEA1 (Enzo Life Sciences, Plymouth Meeting, PA), mouse monoclonal anti-EEA1 (BD Biosciences, Franklin Lakes, NJ), mouse monoclonal anti-human LAMP1 (CD107a; BD Biosciences) and rabbit polyclonal anti-human LAMP1 (Thermo Fisher Scientific, Waltham, MA). HRP-labeled secondary antibodies were from Amersham [53] or Sigma-Aldrich (St Louis, MO) and fluorescently labeled secondary antibodies from Jackson Immunoresearch Laboratories (West Grove, PA). Wortmannin, nocodazole, brefeldin A, paraformaldehyde, glutaraldehyde, filipin, diaminobenzidine (DAB) and *o*-dianisidine were from Sigma-Aldrich (St Louis, MO).

We obtained EGFP-RAB5 and EGFP-RAB7 from Marino Zerial (Dresden, Germany), mRFP-RAB5 and mRFP-RAB7 from Ari Helenius (Zurich, Switzerland), EGFP-RILP from Cecilia Bucci (Lecce, Italy), HRP-LAMP1 from Matt Russell (Boulder, Colorado), and dMYC-SNX16 from Wanjin Hong (Singapore, Singapore). SNX16 was introduced into pEGFP-C2 or fused with monomeric RFP or Venus, kindly provided by Atsushi Miyawaki (Wako City, Saitama, Japan). CD63-expressing constructs were a kind gift from Cynthia Leifer (Ithaca, NY).

### Microscopy

Immunofluorescence microscopy has been described [54]. When indicated, cells analyzed by immunofluorescence microscopy were fixed with glutaraldehyde [55]. Pictures were captured using a Zeiss Axiophot wide field microscope equipped with a Zeiss 63x Plan-NEOFLUAR objective, a Leica AS MDW wide field microscope with a Leica 63x Plan-APOCHROMAT glycerol immersion objective, a Leica AF6000 LX wide field microscope with a Leica 100x Plan-APOCHROMAT oil immersion objective, or a Leica TCS SP2 AOBS confocal microscope equipped with a Leica 100x Plan-APOCHROMAT oil immersion objective. Time-lapse video microscopy was as described [30]. For 2-channel video microscopy, acquisition was at a frame rate of 1 fps, single channel image acquisition was at 5**–**6 fps. All movies show a time sequence of 30 sec, accelerated 5-fold (movie length of 6 sec). The distribution of HRP-LAMP1 was revealed cytochemically with DAB as a substrate and visualized by phase contrast light microscopy [28], [29]. Sample preparation for electron microscopy was described previously [56], [57].

For quantification of colocalization, 3D image analysis was carried out with Imaris software (Bitplane, Zurich, Switzerland). Briefly, image stacks obtained by confocal microscopy were reconstructed with Imaris into 3D “surface objects” for each channel. Colocalization of “surface objects” from different channels was determined, and expressed as a percentage of the volume of the LAMP1 3D reconstruction. For quantification of Venus-SNX16-positive structure dynamics, the net movement was determined with ImageJ software by subtracting the first frame from the last frame of the image sequence — particle tracking was not feasible at high Venus-SNX16 expression levels due to clustering which resulted into visually merged compartments. The mean pixel intensity of the resulting image, showing only structures that had moved, was normalized to the mean pixel intensity of the first frame. Movement at medium or high Venus-SNX16 expression levels were expressed as a percentage of the movement at low Venus-SNX16 levels.

### Other methods

Cells were transfected with FuGene (Roche Diagnostics, Basel, Switzerland) according to manufacturer's instructions. Microtubules were depolymerized with 10 µM nocodazole for 2 h [23], [52]. Early and late endosome fractionation by flotation in a sucrose step gradient was described [23]. Cholesterol was revealed using filipin as described [3], treatment with brefeldin A was described in [27], and LBPA measurement by ELISA was reported in [20].

## Supporting Information

Figure S1 **Immunocytochemistry with anti-SNX16 antibodies.** Untransfected HeLa cells were fixed and analyzed by immunofluorescence microscopy using antibodies against SNX16 and RAB5 (**A**) or LAMP1 (**B**).
(TIF)

Figure S2 **Labeling of cells expressing Venus-SNX16 with anti-SNX16 antibodies.** HeLa cells transfected with Venus-SNX16 were fixed and analyzed by immunofluorescence microscopy using antibodies against SNX16 and LAMP1. The upper panels (**A**) show all channels, the middle panels (**B**) show Venus-SNX16 and anti-SNX16 labeling, and the lower panels (**C**) show anti-SNX16 staining and LAMP1.
(TIF)

Figure S3 **Overexpression of the RAB7 effector RILP clusters SNX16 in the perinuclear region, but not EEA1.** (**A**) HeLa cells transfected with EGFP-RILP were fixed and analyzed by immunofluorescence microscopy using antibodies against SNX16 and LAMP1. (**B**) HeLa cells transfected with EGFP-RILP and mRFP-SNX16 were fixed and analyzed by immunofluorescence microscopy using antibodies against EEA1.
(TIF)

Movie S1 **Venus-SNX16 and mRFP-RAB7 colocalize on late endosomes.** HeLa cells co-expressing Venus-SNX16 (green) and mRFP-RAB7 (red) were analyzed by fluorescence video microscopy. Shown is a detail in the cell periphery. Yellow color in the merged panel (right) indicates colocalization in peripheral late endosomes. Image acquisition was at a frame rate of 1 fps for 30 sec.
(AVI)

Movie S2 **Overexpression of the RAB7 effector RILP re-distributes the majority of SNX16 to the perinuclear, RILP-induced late endosomal cluster.** HeLa cells co-expressing mRFP-SNX16 (red) and EGFP-RILP (green) were analyzed by fluorescence video microscopy (see Fig 2C). Yellow color in the merged panel (right) indicates re-distribution of SNX16-containing endosomes to the RILP-induced late endosomal cluster in the perinuclear region around the MTOC [18], [19]. Image acquisition was at a frame rate of 1 fps for 30 sec.
(AVI)

Movie S3 **Motility of SNX16-containing membranes.** After Venus-SNX16 expression in HeLa cells, the dynamic properties of SNX16-containing endosomes were analyzed by high resolution time-lapse video microscopy (see Fig 6A). Image acquisition was at a frame rate of 6 fps for 30 sec.
(AVI)

Movie S4 **Motility of Venus-SNX16-positive membranes in cells expressing low levels of Venus-SNX16.** HeLa cells with low Venus-SNX16 expression levels were analyzed by time-lapse video microscopy (see Fig 8A). Image acquisition was at a frame rate of 5 fps for 30 sec.
(AVI)

Movie S5 **Motility of Venus-SNX16-positive membranes in cells expressing high levels of Venus-SNX16.** HeLa cells with high Venus-SNX16 expression levels were analyzed by time-lapse video microscopy (see Fig 8B). Image acquisition and analysis were as for movie 4, to allow direct comparison of SNX16 motility.
(AVI)
